# Entering the post-epigenomic age: back to epigenetics

**DOI:** 10.1098/rsob.180013

**Published:** 2018-03-28

**Authors:** Sebastian Bultmann, Stefan H. Stricker

**Affiliations:** 1 Human Biology and BioImaging, Department of Biology II, Ludwig-Maximilian-Universität, BioMedical Center, Grosshaderner Strasse 2, Planegg-Martinsried 82152, Germany; 2 MCN Junior Research Group, Munich Center for Neurosciences, Ludwig-Maximilian-Universität, Biocenter, Grosshaderner Strasse 9, Planegg-Martinsried 82152, Germany; 3 Epigenetic Engineering, Institute of Stem Cell Research, Helmholtz Zentrum, German Research Center for Environmental Health, Neuherberg, Germany

**Keywords:** CRISPR, epigenome-wide association studies, epigenome editing, Cas9, epigenomics, dCas9

## Abstract

It is undeniably one of the greatest findings in biology that (with some very minor exceptions) every cell in the body possesses the whole genetic information needed to generate a complete individual. Today, this concept has been so thoroughly assimilated that we struggle to still see how surprising this finding actually was: all cellular phenotypes naturally occurring in one person are generated from genetic uniformity, and thus are per definition epigenetic. Transcriptional mechanisms are clearly critical for developing and protecting cell identities, because a mis-expression of few or even single genes can efficiently induce inappropriate cellular programmes. However, how transcriptional activities are molecularly controlled and which of the many known epigenomic features have causal roles remains unclear. Today, clarification of this issue is more pressing than ever because profiling efforts and epigenome-wide association studies (EWAS) continuously provide comprehensive datasets depicting epigenomic differences between tissues and disease states. In this commentary, we propagate the idea of a widespread follow-up use of epigenome editing technology in EWAS studies. This would enable them to address the questions of which features, where in the genome, and which circumstances are essential to shape development and trigger disease states.

## 1. Definitions

Our body consists of around 10^13^ individual cells [1], which have been grouped into 411 cell types [2], although this number might be revised soon through the comprehensive utilization of single-cell approaches [3]. Each differentiated cell develops by progressing through one of several cellular lineages while simultaneously decreasing cellular potency. Each cell type possesses its own characteristic transcriptome defined by a small subset of active genes, whereas those genes specific for other cell identities are silenced. Indeed, keeping inappropriate cell identity genes inactive is crucial for the integrity of the body [4–6]. In humans, only two cell types require the complete genome: the totipotent zygote, which is the progenitor of all cells in the body, and the cells of the germ line, which pass the genome on to the next generation. Nevertheless, only a few cell types dispose unneeded but potentially dangerous genetic material (e.g. red blood cells, which lose their nucleus entirely once terminally differentiated [7]). Despite most differentiated cell types are carrying the complete genetic information, they naturally never breach their lineage barrier. Thus, multicellular organisms critically depend on mechanisms able to silence a majority of their genetic information, some of it for a lifetime.

Which processes are causally inducing or protecting *epigenetic phenotypes*, although studied for decades now, is still avidly debated. In the centre of attention lie *epigenomic processes*, reversible marks, modifications and features of chromatin, a macromolecular structure consisting of unmodified and chemically modified nucleic and amino acids found in the nucleus. Chromatin consists first and foremost of DNA itself, whose bases—primarily cytosine residues—can be chemically modified adding information to the DNA sequence [8]. Chromatin also has a protein component, of which histones are the most prominent members. Owing to the variety of possible amino acid residue modifications, and histone variants expressed, histones are the main source of complexity in mammalian epigenomes. Although at least 100 epigenomic features exist, experimental studies usually concentrate on a handful of marks. Commonly profiled marks in respect to gene silencing include methylation of DNA, lysine 9 and 27 of histone H3, as well as the lack of histone acetylation.

However, with respect to their contribution to gene silencing, we face a paradoxical situation. On the one hand, these marks correlate with inactive loci, the enzymes setting these marks are usually necessary for normal animal development, and well-studied model systems exist proving their functionality in some cases [9]: genomic imprinting for DNA methylation [10], Hox gene regulation for H3K27 methylation [11] and repetitive DNA for H3K9 methylation [12]. On the other hand, chromatin modifying enzymes are rarely specific for chromatin marks. They also usually modify large numbers of other proteins [13,14]. Moreover, global changes of canonical chromatin features when comprehensively analysed have often shown to result in surprisingly little consequences for transcriptomes [15–18], disease or even animal development [19–21]. Thus, as far as we know, *epigenomic marks* possess all the prerequisites to control *epigenetic phenotypes*, but probably only a small fraction of those found in the nucleus might indeed possess this regulative power.

## 2. Marks and profiles

Over the last decades, a plethora of methods has been developed to facilitate epigenomic profiling. In principle, these approaches can be subdivided into two categories by the genomic resolution they offer. Single-base resolution approaches are commonly used to map and quantify DNA modifications. Cytosine methylation (mC) can be detected by bisulfite conversion resulting in the chemical deamination of unmethylated cytosine to uracil while methylated cytosine is protected. After PCR amplification, DNA methylation can be mapped and quantified at single-base resolution by massive parallel sequencing or array hybridization [22]. Profiling oxidized derivatives of mC such as hydroxymethylcytosine (hmC), formylcytosine (fC) and carboxylcytosine (caC) is technically more challenging as during bisulfite conversion hmC or fC/caC cannot be distinguished from mC or C, respectively. However, several methods have been developed that circumvent this problem by additional enzymatic conversion steps prior to bisulfite treatment [23–25]. In contrast to the single-base resolution approaches for DNA modifications, chromatin immunoprecipitation sequencing (ChIP-seq) is used to quantify the relative abundance of histone modifications at a genome-wide level. ChIP-seq relies on antibodies to purify cross-linked and sheared chromatin harbouring-specific histone modifications. This step is followed by massive parallel sequencing of the bound DNA. After mapping the individual sequencing reads back to the genome, histone modification occupancy can be determined by scoring the relative abundance of reads mapped to a specific genomic region [26]. Taken together, the above-mentioned techniques made it possible to gain comprehensive insights into the genome-wide localization of epigenetic marks, their relationship with transcription and their cross-correlation.

While being rather cost-intensive in the beginning, recent technological advances in array technology and sequencing have made large epigenome profiling projects feasible. Large consortia such as ENCODE (Encyclopedia of DNA Elements), REMC (Roadmap Epigenomics Mapping Consortium) and IHEC (International Human Epigenome Consortium) are generating comprehensive epigenomic datasets establishing important references relevant to human health and disease [27–30]. Spanning diverse cell and tissue types, these datasets compile information about histone marks, DNA methylation, DNA accessibility and RNA expression that can be used to define regulatory elements and investigate epigenomic differences arising during lineage specification. Additionally, these consortia have established guidelines and protocols for the generation and analysis of epigenomic data vital for reproducibility and comparability among studies (http://www.roadmapepigenomics.org/protocols).

## 3. Epigenome-wide association studies

Sequencing of the human genome 15 years ago triggered a global effort to uncover genetic causes of human disease. Since then, many genome-wide association studies (GWASs) have been conducted to identify disease-causing variants hidden in the human population. Although these efforts have been highly successful in detecting disease-associated variants, these genetic aberrations often lacked meaningful predictive value [31,32]. While this observation initially came as a surprise, it also highlights that most human conditions are strongly influenced by environmental factors. Indeed, lifestyle, physical activity, diet and exposure to environmental hazards might often be more relevant than genetics. Moreover, even monogenetic diseases can be radically influenced by the environment as demonstrated by the analysis of the hereditary cystatin C amyloid angiopathy in the Icelandic population [33]. How environmental effects manifest themselves molecularly remains unclear; however, environmental imprints are detectable in chromatin and thought to have functional relevance [34].

**Figure 1.**
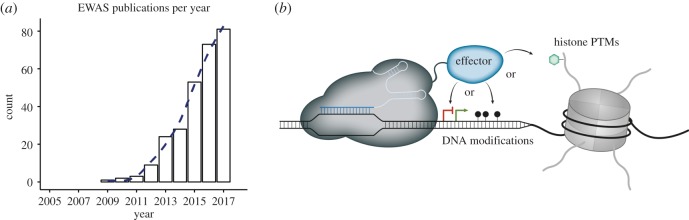
(*a*) Publications on EWAS studies per year (source: PubMed) and (*b*) schematic of dCas9-based epigenome editing approaches.

Technological advances have facilitated the genome-wide examination of epigenetic modifications in the context of disease phenotypes. Akin to GWASs, these epigenome-wide association studies (EWASs) make use of comprehensive cohorts of patients and control groups for large-scale association analysis of epigenomic marks in the context of disease phenotypes. EWAS publications have soared in recent years, primarily focusing on multifactorial disorders including cancer, neurodegenerative and autoimmune diseases (figure 1*a*). Most of these studies have concentrated on DNA methylation largely due to the low amount of tissue required and the cost-effectiveness of detection methods such as Illumina's 450 K array [35]. More recently, the first EWASs examining histone modifications [36], which are considered to be much more dynamic than DNA methylation, have been published. Additionally, disease-associated changes of histone modifications offer the hope of effective treatments due to the variety of inhibitors available, many of which are already in clinical use. Interestingly, while the majority of GWAS hits have been mapped to non-coding and enhancer elements [37], systemic (meta-) analysis for genomic feature association of EWAS hits is still lacking. One reason for this might be that the majority of EWAS data has been produced using Illumina's 450 K arrays, which are inherently biased for promoter and coding regions. Nevertheless, several studies have reported significant association of differentially methylated regions with intergenic regions and DNase I hypersensitive sites [38,39]. With increasing evidence that changes in enhancer signatures are associated with diseases such as cancer [40], a thorough reevaluation of EWAS hits in relation to enhancer sequences would be very informative.

Taken together, EWASs have yielded thousands of epigenomic markers associated with human diseases, but causes of the disease-associated epigenomic changes remain largely unclear, a challenge that has long been also recognized for GWASs [41]. Whereas GWAS hits are being validated using *in vivo* and *in vitro* models of genetic variants, techniques for testing the relevance of individual epigenomic marks have been completely lacking. However, Cas9-based approaches developed in recent years now offer the unique chance to functionally test EWAS hits and to start dissecting cause and effect of epigenomic changes associated with human disease.

## 4. Epigenome editing

First observed in *Escherichia coli*, approximately half of all bacteria (approx. 40%) and nearly all archaea (approx. 90%) are equipped with a sophisticated adaptive defence mechanism called clustered regularly interspaced short palindromic repeats (CRISPR) or CRISPR-associated (Cas) [42]. These systems rely on small CRISPR-RNAs (crRNAs) guiding nucleases to invading nucleic acids. All CRISPR/Cas systems comprise a set of *cas* genes, organized in operons, and a CRISPR-locus harbouring an array of genome-targeting sequences (termed spacers), which are derived from invading foreign DNA and flanked by identical direct repeats. CRISPR/Cas systems are classified into four different types with class II being the best studied and most widely adopted system as a molecular tool [43]. In type II CRISPR/Cas systems, the monomeric protein Cas9 is targeted to DNA by a duplex RNA consisting of the locus-specific crRNA and an invariable *trans*-activating RNA (tracrRNA). By fusing the crRNA and tracrRNA into a single-guide RNA (sgRNA) [44], the RNA-guided endonuclease Cas9 has been adapted as a tool for genome engineering in a great variety of cell types and organisms including human, mouse, fly, worm and zebrafish [45–49]. However, the seemingly limitless potential of CRISPR/Cas does not stop with its utilization as a site-directed nuclease. A universal recruitment platform that, besides other exciting applications, can be used to manipulate epigenetic modifications at defined genomic loci has been generated through inactivation of its endonuclease domain (dCas9).

The principle is as simple as it is elegant. A chromatin modifier is fused to dCas9 and recruited to a chosen genomic site by a specific sgRNA (figure 1*b*). Once the modifier is bound to the target site immediate consequences of the change in epigenomic marks can be monitored. Direct fusions of dCas9 with effectors have already been successfully employed to shed light on the causal relationship between epigenomic marks and levels of transcription. For instance, recruitment of the catalytic core of the human acetyltransferase p300 to promoters or enhancers leads to robust transcriptional activation [50]. Additionally, targeted DNA demethylation within promoter regions, mediated by dCas9-TET1 fusions, results in gene reactivation [48,51]. By contrast, dCas9-mediated recruitment of DNA de novo methyltransferases or histone demethylases to specific gene regulatory regions results in a local repressive epigenetic state and gene silencing [52–55]. These examples highlight the power and feasibility of epigenome editing approaches in elucidating the causality of epigenomic marks. More and more tools are being developed to further exploit the full potential of this novel technique. Besides engineering the dCas9 protein directly, several studies have already demonstrated that the sgRNA can be modified to indirectly recruit effector proteins to a desired genomic locus. MS2 aptamers can be inserted at the tetraloop and stem-loop 2 of the sgRNA which in turn recruit effector proteins fused to MS2-coat proteins [56]. This approach not only allows the recruitment of different epigenetic modifiers to the same locus without mutual interference but it also increases the number of modifiers that can be recruited per Cas9:sgRNA complex [57,58]. An extension of this system using additional motifs for RNA binding proteins (e.g. PP7:PCB or PBS:PUF-domains) has already been shown for dCas9-driven transcriptional activation and genomic visualization approaches [59,60]. Translation of this method to epigenome editing will greatly simplify the simultaneous targeting of multiple genomic loci with different activities. Other challenges, such as defining the correct positioning and the number of sgRNAs required for the desired effects, are currently being assessed in a locus-to-locus manner. Here, systematic studies addressing the principles of efficient sgRNA placement for epigenome editing will be invaluable.

## 5. A blueprint for action

**Figure 2.**
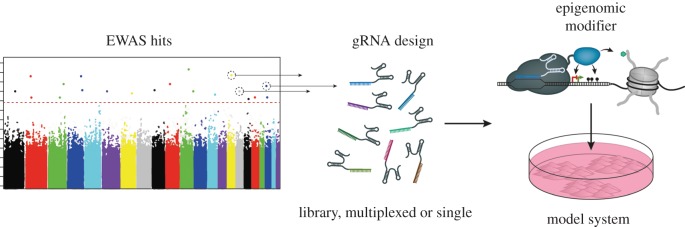
A blueprint for action: validation of EWAS hits by epigenome editing.

Despite the growing amount of profiles and EWAS data available, the functional value of associated hits remains elusive. At least for DNA methylation, a large number of high-quality profiles with disease-associated changes and a CRISPR toolbox to manipulate those have been reported. We propose to enter the post-epigenomic stage by choosing a number of candidate features from the high-quality epigenomic data generated to date and test their relevance by epigenome editing (figure 2). For some researchers, this progression from global analysis to single candidate sites might seem like a step backwards; however, this strategy would also be linked to a progression from descriptive and correlative analysis to functional tests of causality.

Several reviews have already suggested the use of epigenome engineering to validate or screen EWAS hits or profile peaks [9,61]. Testing of causality should not be seen as a separate approach but rather included into the primary study design. Besides high technical standards, uniformity in cell-type composition of the tissue used in the respective study is of utter importance. Most tissues consist of a mixture of cell types, which, when analysed in bulk, yield a mixture of epigenomic profiles resulting in a loss of sensitivity caused by the dilution of disease and cell type-associated signals. Moreover, because epigenomic profiles of the underlying (pure) cell populations are rarely known, slight shifts in their proportions might be mistaken for epigenomic changes. The emerging applications of single-cell epigenomics might resolve these issues to some degree in the future [62]. Today, input heterogeneity is mostly addressed by isolating target populations via (e.g. flow cytometry or laser capture microdissection from donor tissues, as exemplified by recent studies using purified cells of human post-mortem brains to study the association between DNA methylation, ageing and Alzheimer's disease in neurons and glia [63,64]). Performing epigenome profiling on purified cell populations has the added benefit that hits can be directly attributed to specific cell types. A functional EWAS blueprint would not come to an end with the generation of a candidate list of highly significant disease-associated chromatin marks. Instead, an assessment whether the detected alterations are functional drivers of the disease or merely markers would be included. Once disease-associated epigenomic profiles have been determined, a series of epigenome editing tools (e.g. dCas9-TET1, dCas9-DNMT3A) can be employed to remove or establish candidate marks *in vitro*. Essential for this is an experimental system that allows manipulation of the epigenomic mark and modelling of the human disease or phenotype (figure 2). Manipulated cells would then be scored *in vitro* for a disease-associated phenotype. Depending on the phenotype, this approach might be complex, but could also be as straightforward as measuring gene expression changes by qPCR. Candidate epigenomic features could either be tested one by one, several together (using gRNA multiplexing tools) or together in a follow-up epigenomic screen using specially designed libraries of gRNAs. Although a perfect model system might rarely exist—diseases are transformations of organs not cells—certain aspects can often be reproduced *in vitro*. Moreover, the first attempts to transfer epigenome editing into the living animal have already been reported [65,66]. It is possible that some relevant chromatin features might withstand functional analysis *in vitro*, for example, if their effects depend on a very specific genetic background. We think, however, likely incidences of false negative results for some should not discourage from producing causal evidence for many other EWAS hits or profiling peaks.

Although the suggested procedure has not yet been adopted and the functional relevance of thousands of published EWAS hits remains unclear, several publications support the feasibility of editing DNA methylation as well as the functionality for some tested marks [67,68]. Moreover, epigenome editing enables epigenomic manipulation far beyond DNA methylation, while many epigenomic marks remain still to be profiled during development and disease. As such, very few studies have aimed to characterize the alterations of other chromatin marks beside DNA methylation in disease states, the most prominent example being the recently published histone acetylome-wide association study (HAWAS) in autism spectrum disorder [36]. It is currently unknown which epigenomic features will eventually represent a new class of potential targets with the largest impact on gene expression, development and disease. Taken together, epigenome editing offers the best chance of harnessing the immense wealth of information generated by EWASs and a unique opportunity to progress from disease-associated to disease-causing—thus, from epigenomics back to epigenetics.

## Data accessibility

This article has no additional data.
